# Cutaneous Pseudolymphomas With Identical Clinicopathological Features Induced by Tick Bites at Two Separate Sites

**DOI:** 10.1002/ccr3.70782

**Published:** 2025-08-06

**Authors:** Tomoaki Takada

**Affiliations:** ^1^ Sumikawa Takada Dermatology Clinic Sapporo Hokkaido Japan

**Keywords:** dermoscopy, immune response, immunohistochemistry, pathology, pseudolymphoma, tick bites

## Abstract

Identical dermoscopic and histopathologic findings at two distinct tick bite sites demonstrate a stereotypical immune response within a single individual of cutaneous pseudolymphoma, with diagnostic clues including retained cement cones and white reticular lines, even in the absence of visible tick parts.

## 1 Introduction

Tick bites can lead to a wide range of cutaneous reactions, from transient erythema to complex immune responses like granulomatous dermatitis and cutaneous pseudolymphoma (CPL) [1, 2, 3, 4]. CPL is a benign lymphoproliferative condition that mimics cutaneous lymphoma both clinically and histopathologically. It can arise from a variety of external triggers, including arthropod bites, trauma, tattoos, and medications [2, 3]. Among arthropod‐induced CPLs, tick bites are less common but well‐documented causes [3, 4]. Histologically, CPLs show dense lymphocytic infiltrates with variable architecture, including follicular or diffuse patterns, and may display either B‐cell or T‐cell predominance [1, 4]. In tick bite‐associated CPLs, proteinaceous cement material secreted by the tick may be retained in the skin and is often surrounded by inflammatory infiltrates [3, 4]. Immunohistochemistry and molecular testing are often essential to distinguish these lesions from malignant lymphomas [1, 4]. Dermoscopy serves as a valuable diagnostic aid. In CPL, especially when induced by arthropod bites, dermoscopic features such as central crusting, white reticular lines, and linear or serpentine vessels may be observed [5]. These correlate histologically with fibrosis, vascular proliferation, and lymphoid infiltrates. This report aims to demonstrate the reproducibility of clinical and pathological features in tick bite‐induced pseudolymphoma across two separate anatomical sites in a single patient. We describe a rare case of two anatomically separate but histopathologically identical pseudolymphomatous lesions induced by independent tick bites. The striking resemblance across clinical, dermoscopic, histologic, and immunophenotypic features underscores a stereotypical immune response to tick saliva within a single individual [3, 6].

## 2 Case History/Examination

**FIGURE 1:**
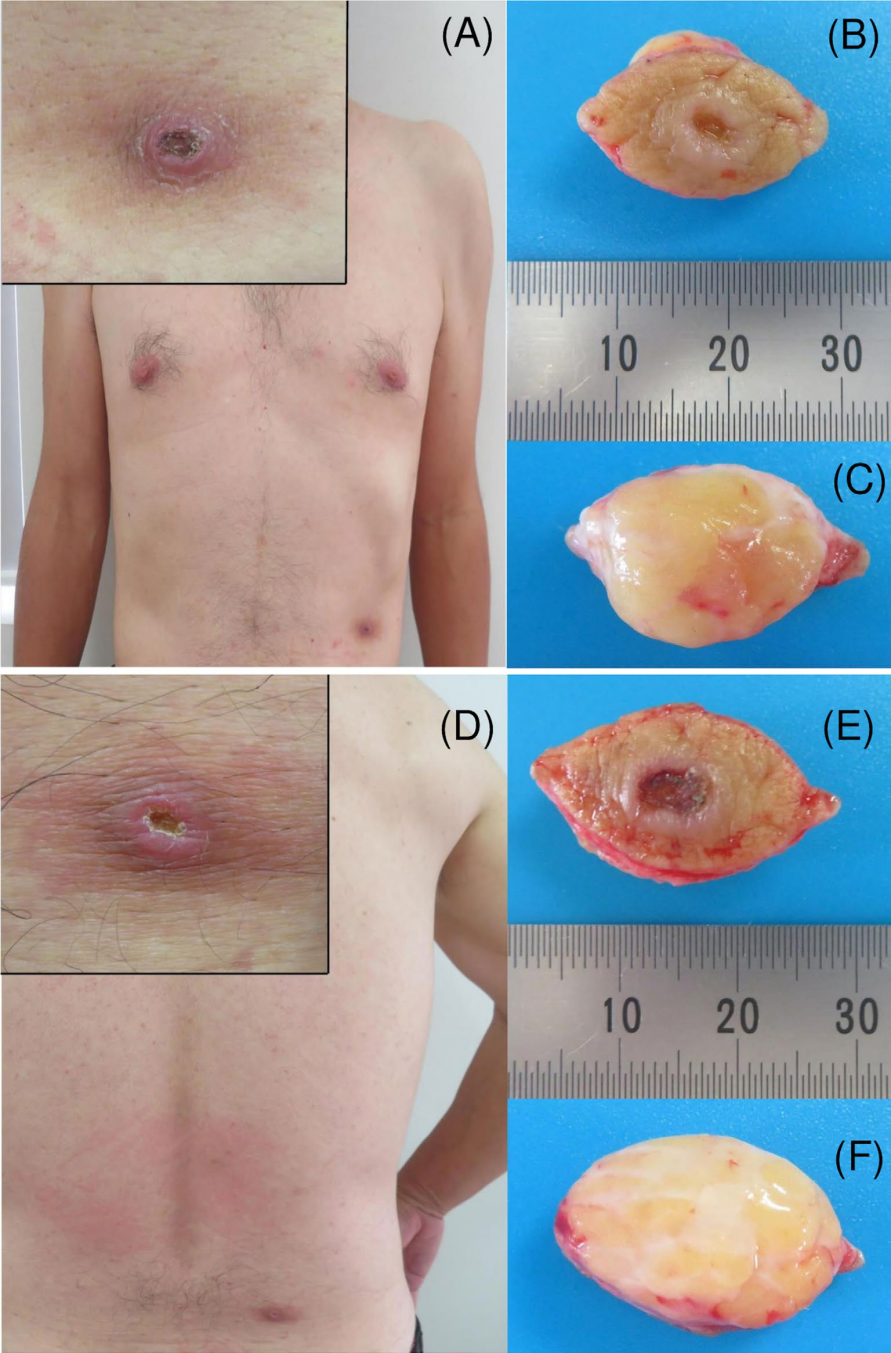
Clinical presentation of the lesions. (A–C) The lesion on the left lower chest; (D–F) the lesion on the right lower back. (A, D) Centrally ulcerated nodular lesions (black boxes indicate magnified areas). (B, E) The excised specimens from the skin surface; (C, F) the reverse sides of the excised lesions.

A 50‐year‐old male forestry worker with no significant past medical history presented with two persistent ulcerated nodules on the left lower chest and the right lower back. The patient reported having brushed off ticks with his hand from both sites the previous day while working in a forested area. He remained asymptomatic and did not seek immediate medical attention. We have reviewed the patient's history and confirmed that he experienced tick bites at ages 35, 42, and 47. However, after 1 month, both bite sites had failed to heal, prompting a dermatological consultation. Clinical examination revealed an 11 × 10 mm ulcerated nodule on the left lower rib region (Figure 1A) and a 12 × 10 mm lesion on the right lower back (Figure 1D). Notably, both lesions displayed central crusted ulcers surrounded by dark red, dome‐shaped nodules with sharply demarcated borders. On palpation, firm subcutaneous nodules were evident, approximately 10 mm in diameter, accompanied by surrounding erythema.

## 3 Differential Diagnosis, Investigations, and Treatment

**FIGURE 2:**
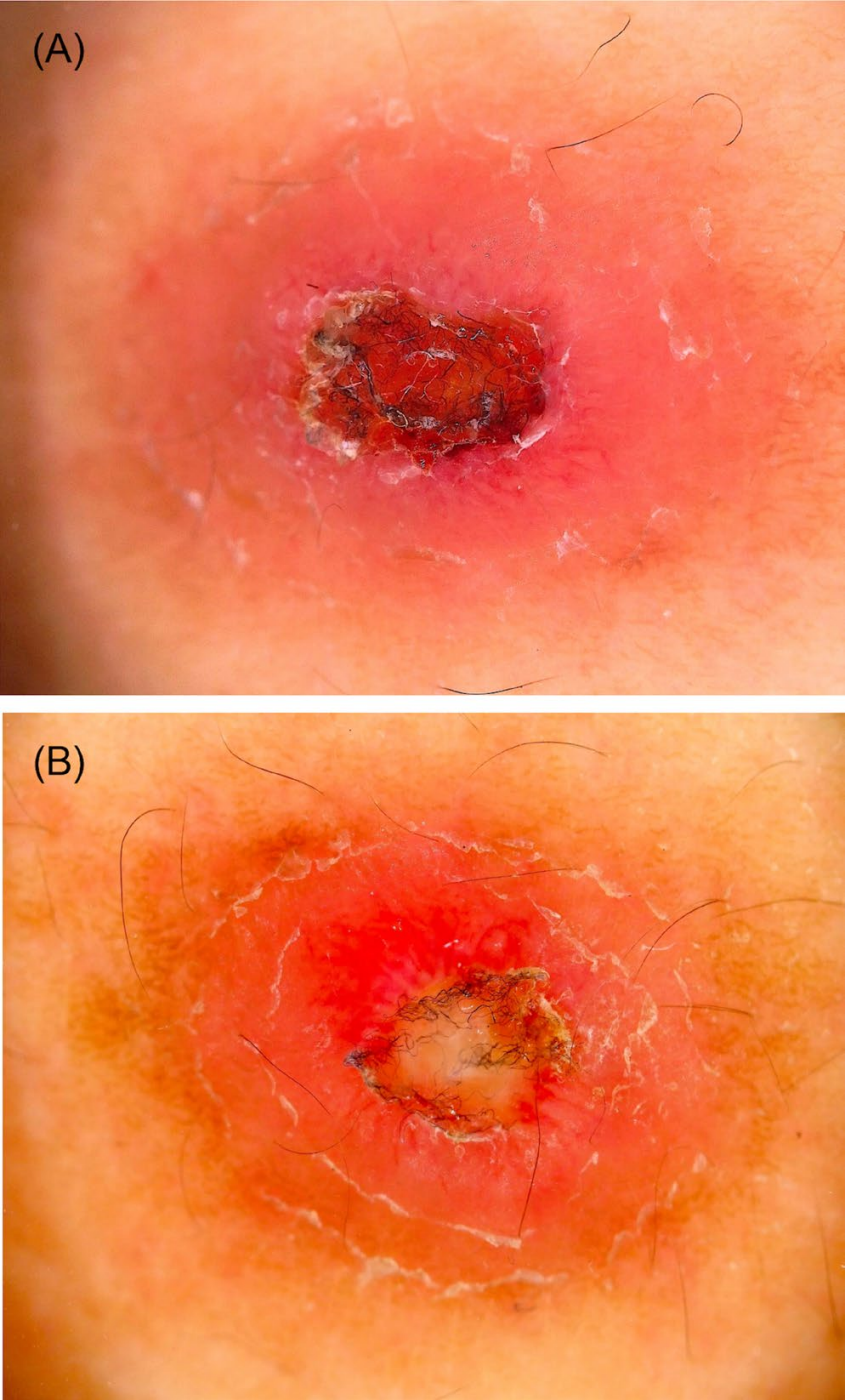
Dermoscopic findings. (A) Corresponds to the lesion on the left lower chest, and (B) to the lesion on the right lower back, matching Figure 1A,D, respectively. Both lesions exhibit a central black crust with a fibrous texture and ulcer base. The peripheral areas show a pink‐to‐red background with white reticular lines and linear vessels. Some shiny white structures are also visible.

**FIGURE 3:**
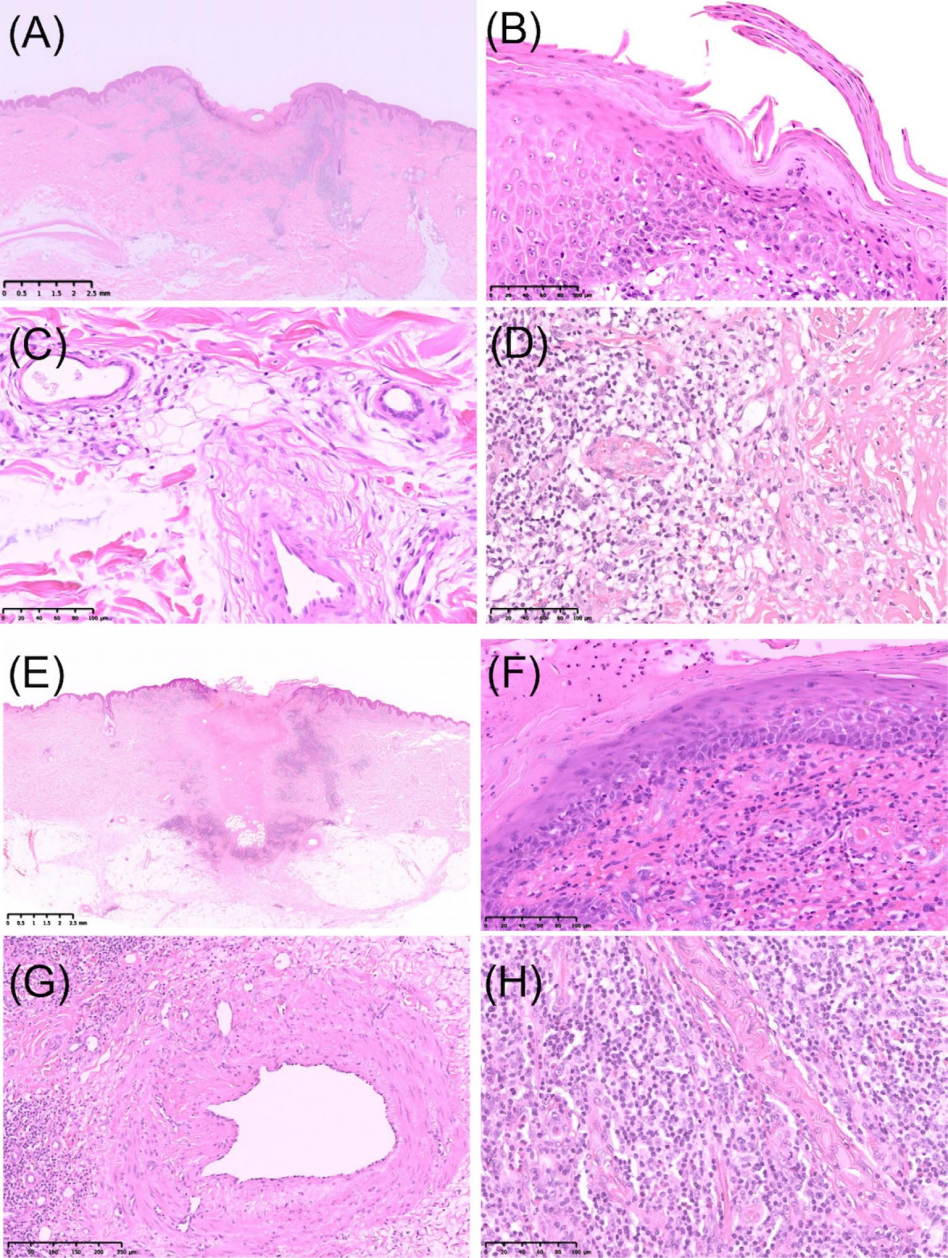
Histopathological findings using hematoxylin and eosin (H&E) staining. (A–D) Correspond to the lesion on the left lower chest; (E–H) to that on the right lower back. (A, E) Overview of the resected specimens showing partial epidermal loss with ulceration. There is dense, band‐like and nodular infiltration of inflammatory cells extending from the upper to the deep dermis. Original magnification: ×5; scale bar: 2.5 mm. (B, F) Subcorneal cement cones and spongiform, blood‐soaked dermis at the ulcer margins. Original magnification: ×200; scale bar: 100 μm. (C, G) Concentric endothelial proliferation and cribriform vessels in the dermis. (C: ×200; scale bar: 100 μm; G: ×100; scale bar: 250 μm.) (D, H) Dermal and subcutaneous sclerosis with dense infiltration of small, bland lymphocytes admixed with histiocytes and eosinophils, particularly around blood vessels. Original magnification: ×200; scale bar: 100 μm.

**FIGURE 4:**
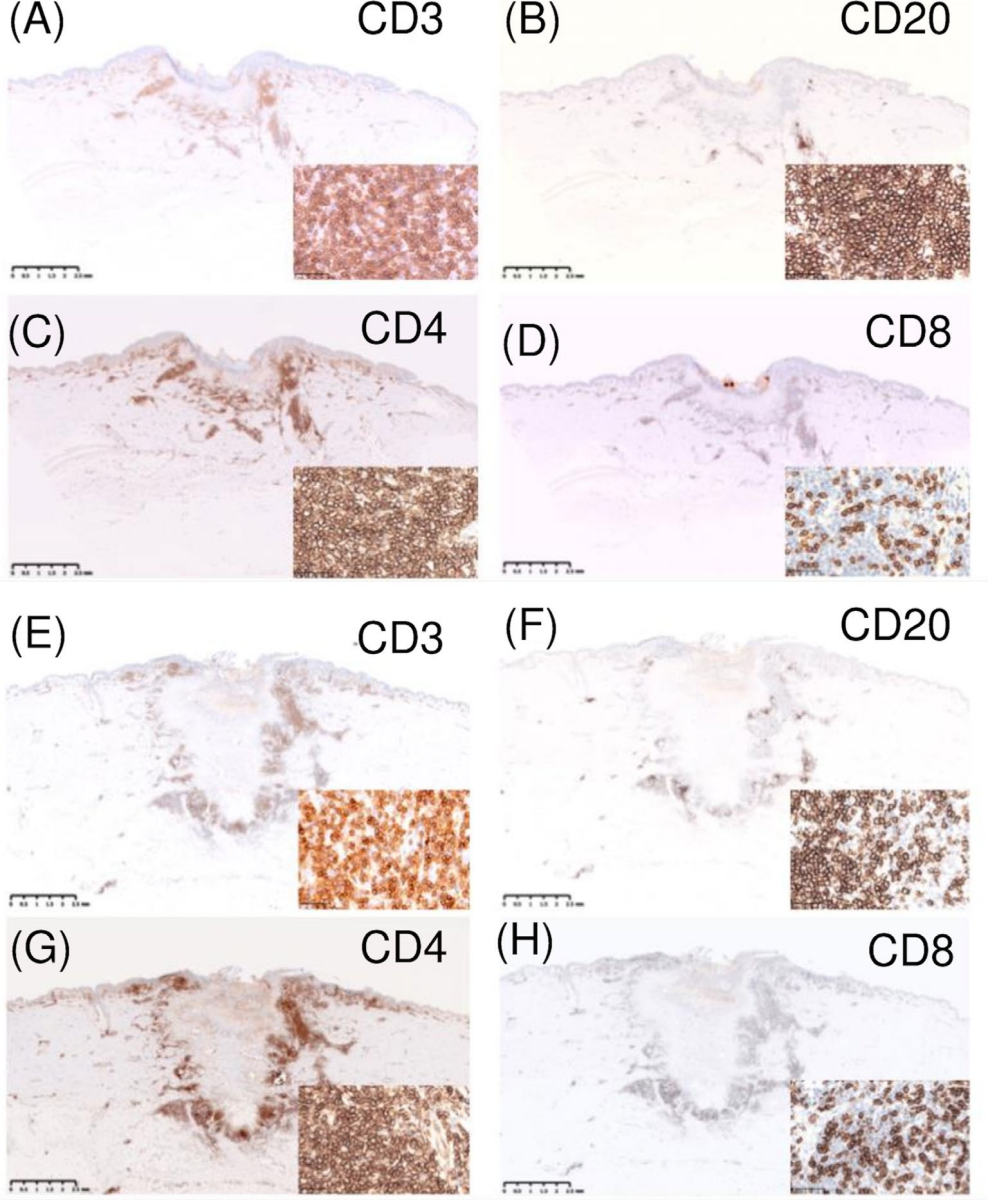
Immunohistochemical staining indicating T‐cell predominant pseudolymphoma. (A–D) Correspond to the lesion on the left lower chest; (E–H) to that on the right lower back. CD4^+^ T cells are more abundant than CD8^+^ T cells. (A, E) CD3; (B, F) CD20; (C, G) CD4; (D, H) CD8. Original magnification: ×5; scale bar: 2.5 mm. Insets: Original magnification: ×400; scale bar: 50 μm.

Dermoscopy of both lesions (Figure 2A,B) showed remarkably similar findings: a central black crust with embedded fibrous foreign material, surrounded by a pinkish‐white background traversed by white reticular lines and irregular linear vessels. Additionally, white streaks and white dots were observed at the peripheral zones. Based on the clinical and dermoscopic appearance, cutaneous pseudolymphoma secondary to tick bites was suspected, and both lesions were excised under local anesthesia (Figure 1B,C,E,F). Histopathological examination (H&E staining) demonstrated almost identical features in both lesions (Figure 3). The epidermis was focally or completely ulcerated and covered by a crust containing amorphous (Figure 3A,E), grayish‐blue to pink material consistent with retained tick cement cones (Figure 3B,F). These cone‐shaped structures extended from the epidermal surface into the upper dermis. The dermis and subcutaneous tissue exhibited localized sclerosis of collagen fibers with surrounding nodular infiltrates of small, cytologically bland lymphocytes, intermixed with histiocytes and eosinophils (Figure 3D,H). In some areas, the ulcer edge was replaced by a disorganized, reticulated network of fibrin, endothelial cells, and collagen. This histologic pattern resembled a spongiform, blood‐soaked dermis (Figure 3B,F). A subset of small vessels exhibited concentric endothelial proliferation and cribriform patterns (Figure 3C,G). Histologically, we did not observe epithelioid cells, foreign body‐type giant cells, or retained mouthparts. Immunohistochemistry revealed a predominance of CD3^+^ T lymphocytes over CD20^+^ B lymphocytes, with CD4^+^ cells outnumbering CD8^+^ cells, supporting the diagnosis of a T cell‐dominant pseudolymphomatous infiltrate (Figure 4).

## 4 Outcome and Follow‐Up

The final diagnosis was cutaneous pseudolymphomas induced by tick bites. The postoperative course was uneventful, with no local infection, fever, or development of new lesions. No evidence of tick‐borne infectious disease was observed during follow‐up.

## 5 Discussion

Cutaneous pseudolymphomas represent benign reactive lymphoid proliferations that can closely resemble lymphomas both histologically and clinically [1, 3]. Their pathogenesis involves chronic antigenic stimulation by exogenous factors such as arthropod bites, including ticks [2, 3]. In the current case, two anatomically separate CPL lesions developed in response to independent tick bites, exhibiting nearly identical histopathological and immunohistochemical profiles. The striking similarity between the lesions suggests that tick saliva elicits a stereotyped immunological milieu, shaped by a complex array of immunomodulatory substances [6, 7, 8].

Emerging evidence has highlighted the role of nonprotein bioactive molecules in modulating host immune responses [7, 8]. These molecules suppress inflammatory pathways by targeting both innate and adaptive immune mechanisms, thereby facilitating the formation of lymphoid aggregates typically seen in tick‐associated CPL. [7, 8] Histologically, both lesions demonstrated dense dermal lymphocytic infiltrates with CD3^+^/CD4^+^ T‐cell predominance, retained cement‐like material, and fibrosis hallmarks described in tick‐induced CPL [3, 4]. There was no evidence of atypia, Pautrier microabscesses, or epidermotropism, which are key features of mycosis fungoides, thus helping to exclude cutaneous T‐cell lymphoma [1]. T‐cell receptor (TCR) gene rearrangement testing was not performed in this case. Among the immunohistochemical markers, Ki‐67 was examined and showed only weak positivity in the infiltrating lymphocytes. However, Bcl‐2, Bcl‐6, and PD‐1 were not tested. In addition, Borrelia‐associated lymphocytoma typically presents as a solitary, bluish‐red nodule in Borrelia‐endemic areas and is characterized by dense B cell and polyclonal plasma cell infiltrates, sometimes with detection of spirochetes by Warthin–Starry staining [6]. These features were absent in our case. Serological tests and specific histochemical stains such as Warthin‐Starry were not performed, as the patient showed no systemic symptoms and the clinical course was not suggestive of Borrelia infection. The dermoscopic features, including central crust, white reticular lines, and linear vessels, were consistent with arthropod bite‐associated pseudolymphoma [5]. When combined with a clinical history of tick exposure and lack of systemic symptoms, these findings strongly supported a benign, reactive lymphoid proliferation rather than a neoplastic process. This case emphasizes the diagnostic utility of clinicopathologic correlation and the importance of recognizing tick bites as a potential cause of CPL. The identification of stereotypical immune responses within a single individual across lesions enhances our understanding of tick‐host immune dynamics and supports the benign, self‐limited nature of the condition. Beyond its diagnostic implications, this case underscores the importance of integrating clinical history, dermoscopy, and immunohistopathology in evaluating atypical nodular skin lesions, particularly in tick‐endemic regions. Future multi‐center studies may help validate the broader applicability of these findings and contribute to the refinement of diagnostic strategies for CPL, particularly in distinguishing it from cutaneous lymphomas.

## 6 Conclusion

This unique case highlights a stereotypical immune response within a single individual's cutaneous pseudolymphoma induced by tick bites at two anatomically separate sites in the same patient, providing valuable insight into the host skin's consistent immune response and the diagnostic utility of clinicopathological correlation.

## Author Contributions

**Tomoaki Takada:** conceptualization, data curation, formal analysis, investigation, methodology, resources, validation, writing – original draft, writing – review and editing.

## Ethics Statement

The author has nothing to report.

## Consent

Written informed consent was obtained from the patient for the publication of this case report and the accompanying images.

## Conflicts of Interest

The author declares no conflicts of interest.

## Data Availability

All data generated or analyzed during this study are included in this article. Further inquiries can be directed to the corresponding author.
